# Urothelial Carcinoma in a 22-Year-Old Female with Angelman Syndrome

**DOI:** 10.1155/2017/9298565

**Published:** 2017-04-11

**Authors:** Jessica Pugh, R. Keith Huffaker

**Affiliations:** ^1^Department of Obstetrics and Gynecology, Quillen College of Medicine, East Tennessee State University, Johnson City, TN, USA; ^2^Division of Female Pelvic Medicine and Reconstructive Surgery, Department of Obstetrics and Gynecology, Quillen College of Medicine, East Tennessee State University, Johnson City, TN, USA

## Abstract

A 22-year-old nulligravid white female with Angelman syndrome was noted to have a 4-month history of premenstrual nausea, vomiting, and abdominal pain. She had an echogenic focus in her bladder noted on ultrasound. She was diagnosed with low grade urothelial carcinoma after cystoscopic evaluation with biopsy and was sent to urology for further treatment. Urothelial carcinoma is rare in individuals younger than age 40. Patients may present with gross hematuria. There is often a delay in diagnosis in younger individuals with different genetic mutations noted upon diagnosis.

## 1. Introduction

Urothelial carcinoma is typically a disease found in individuals older than age 40. Younger individuals with urothelial carcinoma tend to have low grade disease with lower incidence of recurrence and progression. There is often a delay in diagnosis in younger patients due to a low index of suspicion and a lower inclination to perform more invasive testing [1]. Bladder ultrasound is a diagnostic test that can be used in children with gross hematuria [1]. Limited information is available regarding a link between Angelman syndrome and urothelial carcinoma.

## 2. Case

A 22-year-old nulligravid Caucasian female with Angelman syndrome presented with a four-month history of premenstrual nausea and vomiting and abdominal pain. Patient had been hospitalized each of the prior four months due to these symptoms of cyclic nausea, vomiting, and leukocytosis which occurred approximately monthly and were attributed to a stomach virus. Gross hematuria was not reported. There is no family history of urinary tract malignancy. Family history is notable for pancreatic cancer and ovarian cancer in non-first-degree relatives. Her prior surgical history includes teeth extractions and arm surgery. The patient does not smoke, but her father does smoke outside home. An ultrasound of the pelvis was obtained during a hospital admission which showed a 7 mm echogenic focus along the posterior wall of the bladder. Urogynecology then evaluated her as an outpatient. On presentation to urogynecology with her mother (primary caregiver), patient was noted to be physically well developed and well-nourished and in no acute distress in a wheelchair. She had abnormal facies and mentation. General physical and abdominal examinations were normal. Patient was unable to tolerate a full examination in the office; therefore, the decision was made to proceed with an examination under anesthesia and cystourethroscopy. Cystourethroscopy revealed an estimated 1.5 cm papillary cauliflower-like lesion on the right bladder sidewall lateral to the ureteral orifice. Cytology revealed atypical urothelial clusters. Biopsy revealed low grade Ta urothelial carcinoma, negative for invasion of lamina propria, with muscle not present for evaluation. Immunohistochemical stains CK20 and P53 were negative. The pathology specimen was reevaluated and noted as low grade papillary urothelial cancer without identified invasion and without muscularis propria sampled. Patient was referred to urology for further evaluation and treatment. Urology performed a transurethral resection of the bladder tumor which was noted to be 1 cm on a narrow stalk. Complete resection of the bladder tumor was performed with mucosa around the stalk of the bladder tumor removed as well with no evidence of bladder perforation. Final pathology was consistent with noninvasive low grade papillary urothelial carcinoma. Patient will continue to be followed by urology for surveillance cystoscopy under anesthesia every 3 months.

## 3. Discussion

Angelman syndrome is classified as an imprinting disorder. Imprinting selectively inactivates either a maternal or paternal allele. With Angelman syndrome, the maternal allele is silenced. This disorder results from the deletion of chromosome 15q12 which inactivates the UBE3A gene [2]. This gene codes for an ubiquitin ligase that is involved in catalyzing the transfer of activated ubiquitin to target protein substrates. These individuals typically have mental retardation, ataxic gait, seizures, and inappropriate laughter (“happy puppets”) [2]. Prader Willi syndrome is similar; however, the paternal allele is inactivated. These individuals have mental retardation, short stature, hypotonia, profound hyperphagia, obesity, small hands and feet, and hypogonadism [2]. Review of the literature at this time has not shown a link between Angelman syndrome and urothelial carcinoma.

Bladder cancer is the 4th most common cancer in men and the 9th most common cancer in women in the United States [3]. Bladder urothelial carcinoma is typically a disease of older individuals. Most patients are over 60 years old with males being affected more than females. Individuals often present with gross painless hematuria. Other presenting symptoms include dysuria, urinary frequency or urgency, and pain. Most tumors found in individuals less than 40 years old are low grade with an increasing percentage of high grade tumors with increasing age. Younger individuals often have a delay to time of diagnosis of urothelial carcinoma due to a low suspicion of cancer and lower inclination to do more invasive testing. While 1.0–2.4% of urothelial tumors are found in patients younger than 40, only 0.1–0.4% of these tumors are found in patients younger than age 20 [3].

Diagnosis is typically delayed in younger individuals as there is an inclination to try to avoid invasive testing especially in children along with an underestimation of hematuria in these individuals. Bladder ultrasound is an option that should be considered in young individuals with hematuria as both a diagnostic and surveillance tool [1]. Ultrasound can show the typical image of an irregular intravesical invading lesion. Voiding cystourethrogram and urography are options that are less sensitive and often the dye density hides the tumor [4]. Cystoscopy is used for definitive diagnosis as one can biopsy or excise the lesion to allow for pathologic evaluation to determine grade of tumor.

As younger patients tend to have a predominance of low grade, noninvasive disease at time of presentation, molecular characteristics of these tumors have been examined. A review was performed to see if there could be a genetic link between Angelman syndrome and urothelial carcinoma. One study documented that in one low grade noninvasive papillary urothelial carcinoma there were deletions of chromosome regions 7q, 9q, and 15q [1]. This deletion in chromosome 15q could potentially be a link to Angelman syndrome or Prader Willi syndrome as chromosome 15q11-13 is involved in this imprinting disorder; however, this link has not been associated as of yet with bladder cancer. Other studies have looked at potential differences between male and female patients. In animal studies, estrogen has been shown to inhibit growth and development of bladder cancer; whereas, androgens may promote the initiation of bladder cancer [5]. This difference could explain the male predominance of disease. As there are some discrepancies in the literature as to molecular and genetic causes of urothelial carcinoma in younger individuals, more research is needed. No links were found in this literature review between Angelman syndrome or Prader Willi syndrome and urothelial carcinoma.

Treatment of urothelial carcinoma depends on extent of disease and should not be based solely on patient's age. Tumor grade and stage should dictate mode of treatment. The goal of therapy is to prevent progression and recurrence. Bladder ultrasound is a method that can be used as surveillance for recurrence. Patients under the age of 40 who present with tumors >3 cm or with high grade disease are more likely to experience recurrent disease [6]. The five-year cancer specific survival rate has been shown to be 95.2% for superficial disease and 83.3% for invasive cancer with an overall survival rate of 93.3% [7]. Individuals younger than 40 typically have lower rates of recurrence and progression with greater survival rates as they typically have low grade, noninvasive disease.

More research is needed in order to fully understand the differences and similarities between urothelial carcinoma in younger and older patients. This future research can help to determine who would be more likely to develop urothelial carcinoma earlier in life and also helps dictate treatment in younger individuals to prevent recurrence and progression of disease.
